# Implementation of a high cell density fed-batch for heterologous production of active [NiFe]-hydrogenase in *Escherichia coli* bioreactor cultivations

**DOI:** 10.1186/s12934-022-01919-w

**Published:** 2022-09-19

**Authors:** Qin Fan, Saskia Waldburger, Peter Neubauer, Sebastian L. Riedel, Matthias Gimpel

**Affiliations:** Chair of Bioprocess Engineering, Technische Universität Berlin, Ackerstr. 76, ACK24, D-13355 Berlin, Germany

**Keywords:** Metalloprotein, Regulatory hydrogenase, High cell density fed-batch, *Escherichia coli*, [NiFe]-hydrogenase

## Abstract

**Background:**

O_2_-tolerant [NiFe]-hydrogenases offer tremendous potential for applications in H_2_-based technology. As these metalloenzymes undergo a complicated maturation process that requires a dedicated set of multiple accessory proteins, their heterologous production is challenging, thus hindering their fundamental understanding and the development of related applications. Taking these challenges into account, we selected the comparably simple regulatory [NiFe]-hydrogenase (RH) from *Cupriavidus necator* as a model for the development of bioprocesses for heterologous [NiFe]-hydrogenase production. We already reported recently on the high-yield production of catalytically active RH in *Escherichia coli* by optimizing the culture conditions in shake flasks.

**Results:**

In this study, we further increase the RH yield and ensure consistent product quality by a rationally designed high cell density fed-batch cultivation process. Overall, the bioreactor cultivations resulted in ˃130 mg L^−1^ of catalytically active RH which is a more than 100-fold increase compared to other RH laboratory bioreactor scale processes with *C. necator*. Furthermore, the process shows high reproducibility of the previously selected optimized conditions and high productivity.

**Conclusions:**

This work provides a good opportunity to readily supply such difficult-to-express complex metalloproteins economically and at high concentrations to meet the demand in basic and applied studies.

**Supplementary Information:**

The online version contains supplementary material available at 10.1186/s12934-022-01919-w.

## Introduction

In view of global warming and depletion of natural resources, scientists are struggling to look for renewable and environmentally friendly alternative energy systems. Hydrogenases, which are complex metalloenzymes capable of reversibly catalyzing the conversion of H_2_ into protons and electrons, can serve a valuable model for these tasks [1, 2]. Although most hydrogenases are typically inhibited or irreversibly inactivated even by trace amounts of O_2_ or CO, certain O_2_-tolerant [NiFe]-hydrogenases capable of sustaining the catalytic activity in the presence of O_2_, are widely distributed in nature [3, 4]. To date, the most well-characterized O_2_-tolerant [NiFe]-hydrogenases come from *Cupriavidus necator* (formerly *Ralstonia eutropha*) H16, which houses four different types of these hydrogenases (membrane-bound hydrogenase (MBH), soluble NAD^+^-reducing hydrogenase (SH), actinobacterial-like hydrogenase (AH), regulatory hydrogenase (RH) [5, 6]. Over several decades, technical systems that use O_2_-tolerant [NiFe]-hydrogenases have been developed. These systems offer tremendous potential applications in e.g. light-driven H_2_ production [7–9], H_2_-driven biofuel cells [10–12] or H_2_-mediated cofactor regeneration [13–15]. However, at present, their biotechnological applications is still hardly feasible due to the low product yields and long process times by cultivation of the native producers [16–19]. Similarly, the heterologous production of these hydrogenases is challenging due to the complicated maturation process and the high specificity of the required maturation proteins [20]. To improve accessibility of hydrogenases, we recently developed a heterologous production system for [NiFe]-hydrogenases in the robust and genetically tractable production host *Escherichia coli* by the example of the O_2_-tolerant regulatory [NiFe]-hydrogenase from *C. necator* [21].

The RH functions as H_2_ sensor regulating the expression of energy-converting hydrogenases (MBH, SH operons) in the presence of H_2_ [22, 23]. It consists of two heterodimers, each formed by the large HoxC subunit (52 kDa) harboring the H_2_-activating [NiFe(CN)_2_CO]-cofactor and the small HoxB subunit (36 kDa) with three [4Fe4S]-clusters [5, 24]. A truncated version, called RH_stop_ protein, that consists of the single HoxBC heterodimer capable of H_2_-oxidation in vitro, has been widely used for spectroscopic studies [25–29]. Previously, this truncated protein as well as wildtype RH were obtained from batch cultivations of *C. necator* at very low yields of 0.1–1 mg L^−1^ (˂ 0.1 mg g ^−1^) [30–32]. Recently, we achieved by heterologous production in *E. coli* BL21 Gold approx. 300 mg L^−1^ RH in shake flasks by the use of the fed-batch-like EnPresso^®^ growth system [33, 34]. However, despite the high yield the product was inactive due to the absence of the [NiFe] cofactor [33]. The biosynthesis and incorporation of the [NiFe] cofactor into the apo-hydrogenase requires nickel and iron as well as six accessory proteins encoded by the *hyp1* operon [35–37]. Furthermore, we achieved the production of catalytically active RH in aerobically grown *E. coli* BL21 derivatives by co-expressing the *C. necator hyp1* operon as well as the HoxN high-affinity nickel permease and the dedicated HypX maturase and addition of NiCl_2_ [21].

The enzyme-based glucose-releasing EnPresso^®^ medium is based on a typical *E. coli* mineral salt medium supplemented with a non-metabolizable polymer as carbon source. The glucose release from the polymer can be controlled by the amount of enzyme added to the culture, resulting in a quasi-linear increase in cell densities despite permanent glucose limitation [38]. In such growth system proteins are produced under optimal metabolic conditions, thus reducing the risk of incorrectly folding and increasing the portion of soluble proteins, as has been confirmed by the production of different recombinant proteins [38–41]. Small-scale cultivations in the EnPresso growth system e.g. in microwell-plates or shake-flasks are suitable for estimation and optimization of transferable process parameters, however, the yields stay lower compared to benchtop bioreactors due to lower oxygen transfer rates, restricted glucose availability and the impossibility to provide an exponential feed rate [39, 42]. Hence, the next rational step was the development of a high cell density fed-batch process. Generally, *E. coli* can be successfully cultivated in bioreactors up to high cell densities by applying a glucose limited fed-batch mode. In this mode, a highly concentrated glucose solution is continuously fed to the reactor as the growth limiting component, and thus the growth rate can be adapted to the oxygen transfer rate, so that aerobic cultivation conditions can be ensured even at high cell densities [43, 44]. In this study, a rationally designed parallelized fed-batch bioreactor process was developed to produce RH at high cell densities. We believe that the data reported herein provide a reasonable bioprocess development approach for realizing difficult-to-express metalloprotein production to meet the demand for active enzyme in basic and applied studies.

## Materials and methods

### Bacterial strains

*E. coli* strain BQF8RH8 (BL21-Gold [*F*^*–*^*ompT hsdS(r*_*B*_*– m*_*B*_*–) dcm*^+^ *Tet*^*R*^* gal endA Hte*] carrying plasmids pQF8 and pQF18) [21] was used as the production host. The plasmid pQF8 contains the genes encoding the RH structural subunits under control of a P_lac-CTU_ promoter, while the plasmid pQF18 harbors the native maturation genes encoding the auxiliary proteins HypA1B1F1CDEX and the nickel permease HoxN under the control of a P_tac_ promoter [21].

### Growth media

All main cultures were performed using a defined mineral salt medium (MSM), as described recently [41]. Additionally, a macro element solution was prepared as a 10 × stock containing 146 g L^−1^ K_2_HPO_4_, 40 g L^−1^ NaH_2_PO_4_ × H_2_O, 20 g L^−1^ Na_2_SO_4_, 25 g L^−1^ (NH_4_)_2_SO_4_, 5 g L^−1^ NH_4_Cl, 10 g L^−1^ (NH_4_)_2_-H-citrate and separately autoclaved for further use in the feeding solution.

For preparation of precultures LB medium (5 g L^−1^ yeast extract, 10 g L^−1^ tryptone, 5 g L^−1^ NaCl for preculture 1) and EnPresso^®^ B medium (EnPresso GmbH, Germany, for preculture 2) were used. For selection, 25 µg mL^−1^ chloramphenicol and 25 µg mL^−1^ kanamycin were added to all cultures.

### Pre-experiments in 24 deepwell plates

To test cell growth and RH expression in MSM under different nickel or iron concentrations, a 24 deepwell flat-bottom OxoDish^®^ (OD24) plate (PreSens Precision Sensing GmbH, Germany), was used for the pre-experiments. The plate is equipped with oxygen sensors at the bottom of each well, which allows *on-line* measurement of dissolved oxygen concentration (DO). *E. coli* BQF8RH8 was first grown in 50 mL of MSM medium containing 9 g L^−1^ glucose in a 250-mL Ultra-Yield™ flask (UYF; Thomson Instrument Company, USA) sealed with a sterile AirOtop membrane (Thomson Instrument Company, USA) at 37 ℃, 250 rpm for 2 h until an optical density at 600 nm (OD_600_) of approx. 0.6 was attained, and then 3 mL of culture were distributed into each well of the 24 OxoDish plate. Subsequently, 50 µM IPTG for induction of protein expression and different concentrations of NiSO_4_ (0, 30, 50, 100, 500, 1000 µM) together with 0.1 mM FeCl_3_ or different FeCl_3_ concentrations (0, 30, 50, 100, 500, 1000 µM) together with 0.1 mM NiSO_4_ were added to the wells. The cultivations were performed at 30 ℃, 250 rpm (Infors HT, 25 mm amplitude, Switzerland) for 21 h of induction. At the end of the cultivation, 3 mL culture broth from each well were collected in two 1.5-mL tubes followed by centrifugation at 4 ℃ with at 16 000 × *g* for 10 min. The cell pellets were stored at -80 ℃ for further analysis.

### Preculture and inoculation conditions for the fed-batch bioreactor cultivations

To ensure a sufficient cell density for inoculation of the bioreactor, a two-step preculture was used. For the 1^st^ pre-culture, 25 mL LB medium in a 125-mL UYF (Thomson Instrument Company, USA) sealed with a sterile AirOtop membrane (Thomson Instrument Company, USA) were inoculated with scratch of colonies from a fresh LB agar plate and incubated for 6 – 8 h at 30 ℃ and 200 rpm (Infors HT, 50 mm amplitude, Switzerland). Next, 1 mL of the pre-seed LB culture was used to inoculate 150 mL EnPresso B medium supplemented with 3 U L^−1^ reagent A according to the manufacturer’s instructions (EnPresso GmbH, Germany). The fed-batch like preculture was performed in a 1000-mL single-use polycarbonate sensor flask (SFS-HP5-PSt3, PreSens Precision Sensing GmbH, Germany), which allows *on-line* monitoring of pH, DO and biomass. The culture was shaken at 30 ℃ and 200 rpm for 20 h reaching an OD_600_ of about 7 (Additional file 1: Fig. S1). Afterwards, the bioreactor was inoculated with the fed-batch like preculture to an initial OD_600_ of approx. 0.2. To prevent foam formation, 0.01% (v v^−1^) sterile antifoam 204 (Sigma-Aldrich, Germany) was added to all precultures.

### Bioreactor fed-batch culture conditions

The high cell density fed-batch fermentation for heterologous RH production was performed in a 3.7-L bench-top bioreactor with 2 L working volume (KLF2000, Bioengineering AG, Switzerland) with following parameters: the temperature was set to 30 ℃ and 18 ℃ before and after induction, respectively, pH was kept at $7.0 \pm 0.2$ by controlled feeding of 25% (v v^−1^) ammonia solution. The bioreactors were equipped with two six-blade Rushton impellers mounted with a distance of 1.5 times the stirrer diameter. The initial stirring speed and air flow were set to 400 rpm and 0.05 vvm, respectively. In order to maintain the DO value above 20% during the fed-batch cultivation, both parameters were stepwise increased manually until their maximum (1,200 rpm, 2 vvm). Foaming was controlled by manual pulse additions with 0.1 mL antifoam when foam appeared.

The fed-batch cultivations started with an initial batch phase in 2 L MSM medium. After glucose depletion (as indicated by zero residual glucose and a sharp increase in DO), the external glucose feeding was initiated exponentially at a specific growth rate (*µ*_*set*_) set to approx. 70% of *µ*_*max*_ in the batch phase according to Eq. 1.

1 $$F\left(t\right)={F}_{0}*{e}^{{\mu }_{set}*t}$$

The initial feed rate *F*_*0*_ (L h^−1^) was calculated according to Eq. 2. The biomass concentration ($X$) was estimated from a previous correlation of OD_600_ with cell dry weight (CDW) values. One unit of OD_600_ corresponds to a cell dry weight of 0.3 g L^−1^ [45]. The specific growth rate (*µ*) was calculated for the period between two consecutive OD_600_ measurements and fitted with the best spline. The biomass/substrate yield *Y*_*x/s*_ was calculated from the batch phase with the initial glucose concentration (S). *S*_*i*_ represents the glucose concentration of the feed solution, and *X*_*0*_ and *V*_*0*_ are the biomass concentration and culture volume at the start of the fed-batch phase, respectively.

2 $${F}_{0}=\frac{{\mu }_{set}}{{Y}_{x/s}*{S}_{i}}\left({X}_{0} {V}_{0}\right)$$

The feeding solution consisted of 650 g L^−1^ glucose supplemented with 10% (v v^−1^) macro element solution and 0.2% (v v^−1^) trace element solution, as well as 1 g L^−1^ thiamine. When a stirring speed of 1,200 rpm and aeration rate of 2 vvm were reached (corresponding to an OD_600_ of about 75), RH production was induced by addition of 150 µM IPTG per OD_600_ of 75. In parallel 0.3 mM NiSO_4_ and 1.5 mM FeCl_3_ were added per OD_600_ of 75. After induction, the feeding rate was kept constant and eventually decreased to avoid anaerobic growth or overflow metabolism. At the beginning of induction, the inducer was also added to the feeding solution at a concentration of 150 µM and constantly fed to the bioreactor with a total additional IPTG of approx. 90 µmol per 2 L bioreactor culture. The production phase was carried out for 132 h at 18 ℃ under glucose-limited conditions. During the whole fed-batch cultivation, 4 mL of 1 M MgSO_4_ was added aseptically to the bioreactor with each OD_600_ increase of ~ 20.

### Sampling and analytical methods of the bioreactor fermentation

Before induction, sampling was performed every 2 h, whereas sampling was carried out in larger time intervals after induction. At every sampling point, the OD_600_ was measured manually in duplicates with a spectrophotometer (Ultraspec 3300, GE Healthcare, USA) at a dilution (in 0.9% NaCl_aq_) in a measurement range of 0.2–0.8.

For CDW determination, duplicates of 2 mL aliquots of the culture were harvested in pre-weighted 2-mL tubes by centrifugation (21,500 × *g*, 4 ℃, 10 min). The pellets were washed under the same conditions with 0.9% NaCl_aq_ to remove residual culture medium. After centrifugation, the washed pellets were dried at 80 ℃ for 24 h and the CDW was determined by weighing the dried cell-containing tubes. The supernatants were analyzed with a Cedex Bio HT Analyzer (Roche Diagnostics International AG, Switzerland) using test kits for glucose, Mg^2+^, ammonia, acetate and iron (Glucose Bio HT, Magnesium Bio HT, NH3 Bio HT, Acetate V2 Bio HT, Iron Bio HT).

Additionally, 1 mL broth samples were collected at the selected sampling times for total protein analysis. The samples were centrifuged (21,500×*g*, 4 ℃, 10 min), the supernatant discarded and the pellet stored at − 80 ℃ until further analysis. For soluble RH purification, cells were harvested from 20 mL culture samples followed by centrifugation at 8,000×*g*, 4 ℃ for 10 min (Eppendorf, Germany) at different times after induction and pellets were stored at  − 80 ℃.

Off-gas data performed with a BlueInOne_FERM_-sensor gas analyzer for parallel measurement of O_2_ and CO_2_ concentrations (BlueSens gas sensor GmbH, Germany) was recorded for both bioreactors during the fed-batch cultivations, and served for the determination of the oxygen uptake rate (*Q*_*O2*_), carbon dioxide production rate (*Q*_*CO2*_), respiration coefficient (*RQ*) and the volumetric oxygen transfer coefficient (*k*_*L*_*a*) based on the gas mass balance.

### RH purification

For total protein analysis, the pellets were resuspended in 2 × SDS sample buffer normalized to an OD_600_ of 25 and heated at 95 ℃ for 20 min. After cooling and centrifugation 12 µL of the SDS-denatured samples were separated in 12% PAA gels. Subsequently, proteins were transferred onto a PVDF membrane (0.45 µm pore size, Carl Roth, Germany) by semi-dry blotting in a Transblot Turbo Transfer system (Bio-Rad, Germany) at 1.3 A/25 V for 30 min. Detection of Strep-tagged HoxB was carried out as described previously [33, 34].

For soluble protein purification cell pellets were resuspended in 4 mL buffer A (100 mM Tris–HCl, pH 8.0, 150 mM NaCl) per g wet cells supplemented with 1 g L^−1^ lysozyme and 1 mM PMSF. The cells were disrupted by sonicating using the UP200S sonicator (Hielscher Ultrasonics GmbH, Germany, 30 s on/off, 7 mm sonotrode diameter, 60% amplitude) for 2.5 min per 2 g of wet cell weight. The cell-free extracts were centrifuged at 8,000×*g*, 4 ℃ for 1.5 h (Eppendorf, Germany) and the supernatant was collected (SE) and immediately applied to Strep-Tactin Superflow columns (IBA, Göttingen, Germany) for soluble RH purification. RH purification and quantification were carried out as previously described [21, 33, 34].

### Western blotting

Western blotting was performed as described recently [33]. For soluble and insoluble protein analysis, 100 µL aliquots lysate suspension after sonication were centrifuged at 16,000×*g*, 4 ℃ for 45 min. The supernatant was transferred into a fresh 1.5-mL tube (soluble fraction) and mixed with 100 µL 2 × SDS sample buffer, whilst the pellet containing insoluble proteins and cell debris was resuspended in 200 µL 2 × SDS sample buffer. Afterwards, the samples were incubated at 95 ℃ for 10 min or 20 min for soluble and insoluble fractions, respectively. 15 µL of each sample were applied for SDS-PAA gel and subsequent Western blot analysis.

## Results and discussion

### Metal effect on cell growth and active RH production in glucose MSM

Previously we used the fed-batch like EnPresso system to screen essential process parameters, e.g. production strain, inducer concentration, production temperature or metal ion supplementation, required for active RH production in deepwell plates or shake flasks [21, 33]. This guarantees that the same cultivation mode (fed-batch mode) is used for small-scale optimization and for large-scale bioreactor cultivation. However, numerous studies have shown that high nickel supplementation has a significantly more negative effect on cell viability in MSM compared to complex media [46–48]. So before proceeding with bioreactor cultures, we analyzed the effects of nickel and/or iron additions on cell growth and active RH production in glucose MSM cultures.

**Fig. 1:**
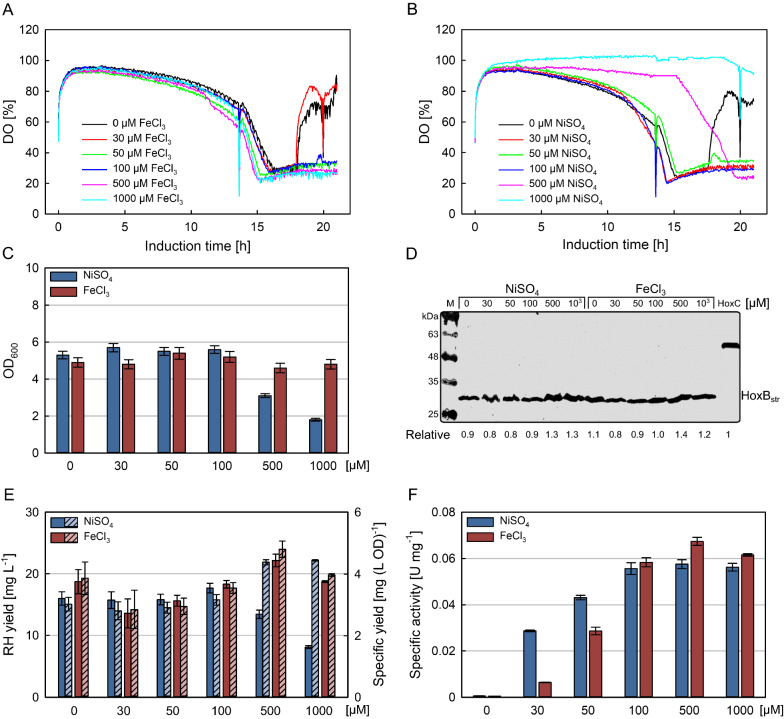
Screening of nickel and iron concentrations in mineral salt medium. Cells were grown in mineral salt medium with 9 g L^−1^ glucose. After induction with 50 µM IPTG, 3 mL culture were distributed in 24-deepwell plates at 30 ℃, 250 rpm and supplemented with different nickel or iron concentrations. 0.1 mM FeCl_3_ was added for nickel screenings while 0.1 mM NiSO_4_ was added for iron screenings. For RH detection 3 mL cells were normalized to an OD_600_ of 30 with 50 mM KH_2_PO_4_/K_2_HPO_4_ buffer pH 7.0 and sonicated followed by centrifugation. 12 µL soluble protein sample was separated on 12% polyacrylamide gels followed by Western blotting where HoxB from RH was detected using anti-Strep tag antibody. 1 µg HoxC_str_ was used as control. **A**, **B** profiles of online dissolved oxygen concentration (DO) of the cultures after induction. **C** Final OD_600_ of cultures, **D** Western Blot (WB) analysis of soluble crude extracts. Bands were quantified relative to the control HoxC_str_ using ImageJ as shown below the blots. **E** soluble RH yields (without slash) and specific yields (with slash) as quantified from WB analysis, **F** specific activities measured from soluble crude extracts. All measured ODs and yields are the mean of two independent technical replicates

Thus, we performed deepwell plate cultivations of *E. coli* strain BQF8RH8 in 3 mL MSM with 9 g L^−1^ glucose supplemented with different concentrations of NiSO_4_ or FeCl_3_ as described in Materials and methods. The dissolved oxygen concentration (Fig. 1A and B) was measured *on-line* over the entire induction time as an indicator for cell growth. In addition, the final OD (Fig. 1C), total RH (Fig. 1D and E) and its activity in the soluble protein extract (Fig. 1F) were determined at the end of cultivation. With increasing cell growth, more oxygen is required, which leads to a decrease in the DO level. In case of iron addition, no differences in oxygen consumption irrespective of the iron concentration were observed (Fig. 1A), suggesting no negative impact on cell growth. On the contrary, the increase of the DO level after about 17 h at 0 and 30 mM FeCl_3_ (Fig. 1A) indicates a drop in the metabolic activity of the cells that might be the consequence of an iron deficiency caused by an increased iron demand due to RH production. While the addition of up to 100 µM NiCl_2_ lead to a similar decrease of the DO level as in the case of FeCl_3_, addition of 0.5 or 1 mM NiCl_2_ resulted in a significantly reduced DO consumption, which indicates a significant reduction of cell growth or prolonged cellular adaptation of the strain in mineral salt medium (Fig. 1B). Interestingly, without additional nickel, a rise in the DO level is also observed after about 16 h of induction (Fig. 1B), indicating a limitation of nickel analogous to the iron limitation. In both cases, the results of the DO curves are confirmed by the final ODs (Fig. 1C). Similarly, none of the tested iron or nickel additions affected the specific RH production (Fig. 1D, E), thus higher nickel concentrations resulted in a significant decrease in total RH yield due to the reduced growth (Fig. 1E). However, increasing metal concentrations of up to 0.1 mM nickel or 0.5 mM iron had a positive effect on the specific RH activity, which ceased to increase at higher metal ion concentrations (Fig. 1F). This is in line with previous studies on recombinant [NiFe]-hydrogenase production in *E. coli* showing the highest in vitro activity at 25–30 µM NiCl_2_ in modified mineral salt medium cultures [49–52]. Hence, nickel and iron concentrations of 0.1 mM and 0.5 mM, respectively, were selected for the following bioreactor cultivations.

### High cell density fed-batch cultivation

**Fig. 2:**
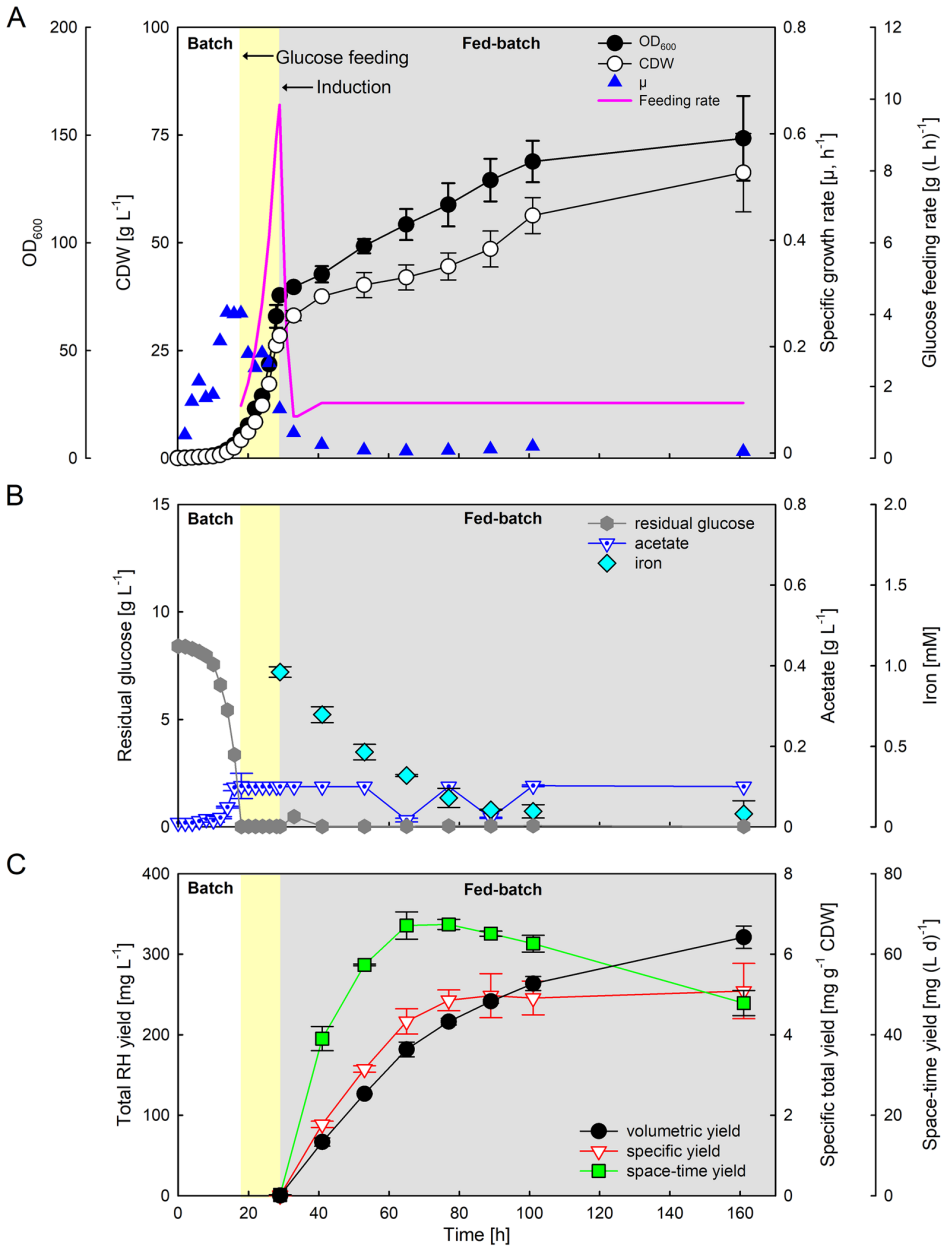
High cell density fed-batch bioreactor cultivation for RH production with *E. coli* BL21-Gold pQF8 and pQF18. **A** Cell growth curve, biomass, specific growth rate and glucose feeding rate. **B** Residual glucose concentration, acetate and iron concentrations measured from culture supernatant. **C** The yields for total volumetric, specific, and space–time RH production were determined from Western blotting analysis after induction. The three phases of the process are shown by different color backgrounds: white batch phase, yellow exponential feeding phase, and gray production phase. All values are the mean of two independent technical replicates from two independent biological replicates

As cultivations in shake flasks with EnPresso B medium cannot meet the possible hydrogenase demand for an industrial application, production in a larger scale and higher cell density is necessary. Thus, we aimed to develop a glucose-limited high cell density fed-batch in a benchtop bioreactor. The cultivations were performed in a 3.7-L lab-scale bioreactor filled with 2 L of MSM minimal medium containing an initial glucose concentration of 8.5 g L^−1^ as sole carbon source. The process was divided into three phases (Fig. 2A): (i) an initial batch phase, followed (ii) by an exponential feeding phase after which the RH production was induced by the addition of IPTG, and (iii) finally the production phase with constant feeding until the end of cultivation. During the 18 h batch phase, the initial glucose was completely consumed, as indicated by offline glucose measurements (Fig. 2B). Whenever the DO dropped below 30% either the stirrer speed or the aeration rate were increased manually to avoid oxygen limitation (Additional file 1: Fig. S2A). At the end of the batch phase the culture reached an OD_600_ of 11 (CDW of 4.2 g L^−1^) and a maximal specific growth rate (*µ*_*max*_) of 0.25 h^−1^ (Fig. 2A). In the batch phase, a biomass yield of 0.5 g biomass g^−1^ glucose was determined, which is slightly higher than the range of the most *E. coli* strains (0.35–0.48 g g^−1^), indicating stress-free growth and effective incorporation of the supplied carbon into biomass [53]. After glucose depletion, an exponential feed with concentrated (650 g L^−1^) glucose solution was started. The feed rate was calculated to ensure a targeted specific growth rate of 0.18 h^−1^ (Fig. 2A). During the whole process, the DO was maintained above 20% by gradually increasing air flow rate or stirring speed to prevent oxygen limitation or anaerobic conditions, as this would lead to the accumulation of acetate or other mixed acid fermentation products that are detrimental to cell growth and recombinant protein production [44, 54, 55]. The exponential feed was stopped after 29 h, when an OD_600_ of approx. 75 was reached (corresponding to a CDW of 28.5 g L^−1^, Fig. 2A). After the exponential feeding phase, the temperature was reduced to 18 °C and RH production induced by IPTG addition. Simultaneously, NiCl_2_ and FeCl_3_ were added to provide sufficient metal ions for RH maturation. A constant feed rate was applied during the production phase, which was gradually decreased due to the lower glucose consumption at 18 °C to avoid acetate accumulation [56–58]. Nevertheless, the cells continued to grow with a specific growth rate below 0.01 h^−1^ until the end of the cultivation, reaching a final OD_600_ and CDW of 150 and 66 g L^−1^, respectively (Fig. 2A). However, neither glucose nor acetate accumulated during the production phase (Fig. 2B). Since addition of IPTG led to a decrease of respiration, the DO level rose and was maintained at 75% during the production phase (Additional file 1: Fig. S2A).

During the cultivation, a final biomass concentration (66.3 g L^−1^ CDW) was reached by consumption of a total of 438.2 g glucose, corresponding to an average yield of biomass per substrate Y_X/S_ of 0.3 g g^−1^ (Additional file 1: Fig. S2B). The obtained OD_600_ of 150 is fivefold higher compared to fed-batch-like EnPresso shake flask cultivations (OD_600_ of 30–40) [21, 33, 34] and even 32-times higher than in batch MSM cultivations performed in deepwell plates (Fig. 1C). It is estimated that the volumetric oxygen transfer coefficient (*k*_*L*_*a*) value in the stirred-tank bioreactor is about threefold higher compared to the 250-mL UYF at 200 rpm (~ 422 h^−1^) [59], thus, enabling aerobic cultivation to high cell densities. As the biomass increased, the gas flow rate was increased up to 2 vvm at a stirring speed of 1,200 rpm (Additional file 1: Fig. S2A). By this way, based on the sensor for exhaust gas analysis, a maximal *k*_*L*_*a* value of approx.1,200 h^−1^ was determined during the growth phase, which is comparable to other *E. coli* cultivations with the same bioreactor system [41]. The respiration quotient (RQ) as a ratio of carbon dioxide produced per oxygen consumed increased after the feed start, and immediately decreased slightly after the induction followed by maintaining at a relatively constant value of about 1 until the end of the cultivation (Additional file 1: Fig S2C). The RQ close to 1 indicates an equilibrium between respiration, cell growth and carbon dioxide production without accumulation of byproducts, e.g. acetate or lactose, which is in line with the acetate measurements (Fig. 2B).

In parallel to induction with IPTG, 1.5 mM FeCl_3_ and 0.3 mM NiCl_2_ were added to provide sufficient metal ions for RH maturation. The iron concentration in the medium decreases steadily as iron is required for the hydrogenase production, and finally levels off at a value of approx. 80 µM, suggesting that sufficient iron is available in the medium (Fig. 2B). To follow the RH production during the production process, samples were collected at different time points after induction and used for the determination of the total RH concentration. The Western blotting analyses indicate that the RH is stably produced and gradually accumulated approx. 320 mg L^−1^ (Additional file 1: Figs. S3 and 2C). Similarly, the specific yield increased to about 5 mg RH g^−1^ CDW in the first 48 h after induction and remained at this level thereafter (Fig. 2C), thereby further demonstrating the stability of the RH production over the entire duration of the process.

### Evaluation of RH yield and activity

**Fig. 3:**
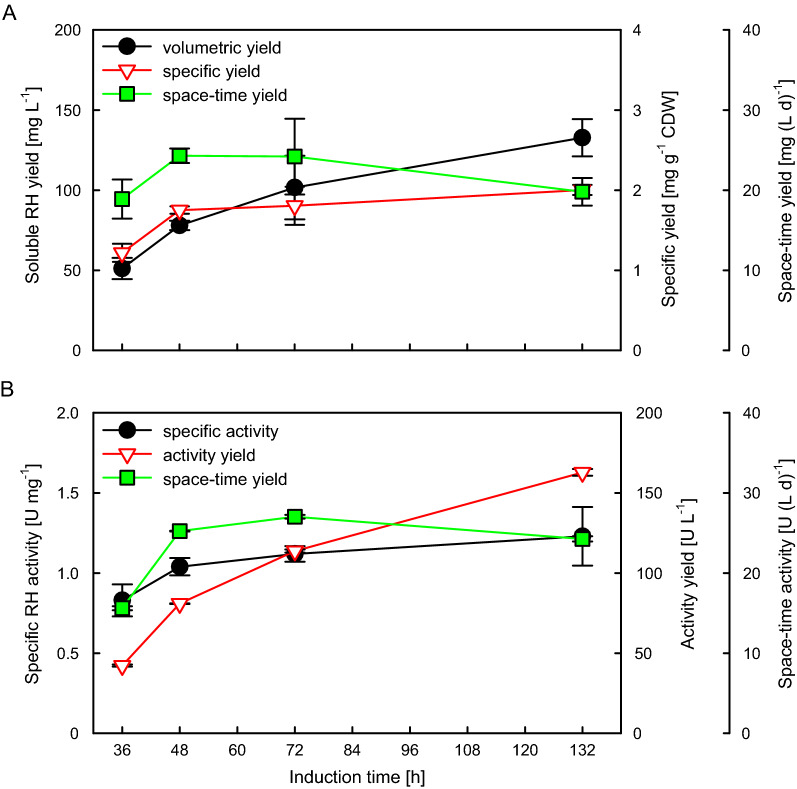
Soluble RH production and activity yield obtained from the fed-batch bioreactor fermentation. 20 mL cells were collected at different induction times from two parallelized bioreactor under the same conditions. Cells were disrupted with sonication on ice (60% amptitude, 7 mm sonotrode, 30 s on/off, 10 min) followed by centrifugation (8000×*g*, 4 ℃, 90 min). The clarified lysates were purified using Strep-Tactin columns (CV of 500 µL). **A** The eluted RH amounts were quantified with SDS-PAGE analysis and **B** the activities of the purified RH samples were measured in vitro. All values are the mean of two independent technical replicates from two independent biological replicates

The soluble RH yield and the enzymatic activity are decisive criteria for assessing the success of a cultivation. To follow the RH production, 20 mL samples were collected at different time points after induction and used for purification of the soluble RH and subsequent measurement of its enzymatic activity. As expected, both volumetric and specific RH yield increased over time (Fig. 3A). While the volumetric yield increased steadily due to the increasing biomass in the reactor and reached a final level of 133 mg L^−1^, the specific yield reaches a maximum of about 2 mg g^−1^ CDW already at 48 h after induction and does not increase significantly further until the end of the cultivation after 132 h of induction (Fig. 3A). Unfortunately, the amount of purified soluble RH corresponds to only about 40% of the total RH yield (Figs. 2C and 3A). When analyzing the protein content of sonication-lysed cells, half of the total RH amount was still found in the insoluble cell fraction (Additional file 1: Fig S4), which has not been observed in shake flask cultivations [21, 33, 34]. The difference may be related to the much higher cell densities and time of cultivation which affect the adaptation of the cellular system in connection to the protein synthesis system and the stress responses. It may be hypothesized that the extracellular cAMP level could be one of the key factors which would affect the expression strength and thus the balance between correctly folding and aggregation of target protein [60]. Besides product formation at high cell densities, expression at low growth rates may provide a positive effect by an increased stress resistance, which could be important in response to environmental changes caused by high cell density cultivations [61]. Nevertheless, previous studies in the measurement of the stringent response ppGpp level during the very slow growth for both recombinant and non-recombinant *E. coli* fed-batch processes, indicate the segregation of a part of the cell population into viable but nonculturable cells at very slow growth rates (˂0.02 h^−1^) [62, 63]. Thus, those may serve as analytical focus points for further improvements in terms of RH activity and solubility in larger scale high cell density bioreactor cultivations.

The space–time yield increases with the volumetric yield, at least at the beginning of the production phase. However, it reaches a maximum of approx. 24 mg (L d)^−1^ after 48 h of induction and then starts to decrease steadily (Fig. 3A). Nevertheless, the soluble protein yield is still more than 130-fold higher than the RH yield obtained from bioreactor cultivations of the native producer *C. necator* [30] and even several thousand-times higher than that of the *Pyrococcus furiosus* [NiFe]-hydrogenase heterologously produced in *E. coli* [49], further demonstrating the value of our cultivation strategy.

Similar to the increase in protein yield, the specific and absolute RH activity increase and reach final values of 1.23 U mg^−1^ and 160 U L^−1^, respectively (Fig. 3B). The activities and yields achieved were 1.3 and 160 times higher than those obtained from *C. necator* H16, respectively. [30]. However, compared to our recently reported data from shake flask cultivations the specific RH activity is about 2.5-fold lower [21]. The specific activity is proportional to the amount of cofactor formed and incorporated into the apo-enzyme, thus it is possible that the RH apoprotein cannot receive cofactors as efficiently as the enzyme produced in shake flasks, presumably due to different medium used. In deepwell plate experiments with MSM medium (Fig. 1) or EnPresso shake flask cultivations [21], the highest RH activity was achieved by addition of 0.1 mM NiSO_4_ at an OD_600_ of 5 or 8, while in the bioreactor cultivation 0.3 mM NiSO_4_ was added only once at an OD_600_ of 75 (Fig. 2). Thus, the lower specific RH activity obtained in the bioreactor cultivations might be attributed to the lower concentration of added nickel relative to the higher cell densities, resulting in insufficient nickel being delivered to the cells. Nevertheless, the production of soluble RH is significantly higher in the bioreactor, thus compensating for the lower specific activity compared to the shake flasks.

## Conclusion

In this study, the scalability and robustness of the heterologous RH production process from small-scale cultivation to laboratory-scale bioreactors was successfully demonstrated. The high cell density fed-batch cultivation resulted in a soluble active RH yield of more than 130 mg L^−1^ and as high as 320 mg L^−1^ for total RH titers, based on a pure glucose limiting feeding strategy. Our results are promising as they show that active RH production is not disturbed by scaling effects, demonstrating the robustness of the hydrogenase production process when providing reproducible growth conditions at different scales. However, a limitation of the current scale-up study is that the highest specific RH activity achieved in previous shake flask scales [21] could not yet be maintained in the bioreactor scale, suggesting potential for further optimization, e.g. in media requirements. In addition, further research will be necessary to elucidate whether the inducer IPTG concentration enables to satisfy the induction behavior of individual cells at high cell density cultures at both macroscopic and microscopic levels, as it can trigger stress responses in *E. coli* leading to pre-induced cells remaining induced and non-induced cells. Nevertheless, we strongly believe that the data reported herein qualitatively and quantitatively provides a reasonable approach for the development of bioprocesses for the production of difficult-to-express metalloproteins to meet the needs in basic and applied studies. The developed bioreactor cultivation system for the production of active [NiFe]-hydrogenases, provides a good opportunity for an economical supply of such metalloenzymes in high concentrations, facilitating basic and applied research on these proteins as well as their potential industrial application. Furthermore, there are also broader implications for the future development of biotechnological production systems for heterologous functional complex heteromeric metalloproteins.

## Supplementary Information

**Additional file 1: Figure S1**. Profiles of dissolved oxygen concentration, pH and biomass of the 2nd preculture in EnPresso B medium. **Figure S2**. Supplementary information of the fed-batch bioreactor cultivations, **Figure S3.** Western Blot analysis of total RH production during the fed-batch bioreactor fermentation, **Figure S4**. Soluble and insoluble fractions of RH with Western blotting analysis.

## Data Availability

All data generated or analyzed during this study are included in this published article [and its supplementary information files].
