# Supplementary material for: Implementation of a high cell density fed-batch for heterologous production of active [NiFe]-hydrogenase in Escherichia coli bioreactor cultivations
Source: Microb Cell Fact. 2022 Sep 19;21:193. doi: 10.1186/s12934-022-01919-w (PMC9484157; doi:10.1186/s12934-022-01919-w)
Supplement: Supplementary file 1 — Additional file 1: Figure S1. Profiles of dissolved oxygen concentration, pH and biomass of the 2nd preculture in EnPresso B medium. Figure S2. Supplementary information of the fed-batch bioreactor cultivations, Figure S3. Western Blot analysis of total RH production during the fed-batch bioreactor fermentation, Figure S4. Soluble and insoluble fractions of RH with Western blotting analysis. [file 12934_2022_1919_MOESM1_ESM.docx]

***Supplementary Material***

**Implementation of a high cell density fed-batch for heterologous production of active [NiFe]-hydrogenase in *Escherichia coli* bioreactor cultivations**

Qin Fan^1^*, Saskia Waldburger^1^, Peter Neubauer^1^, Sebastian L. Riedel^1^ and Matthias Gimpel^1^*

^1^ Technische Universität Berlin, Chair of Bioprocess Engineering, Ackerstr. 71-76, ACK24, D-13355 Berlin, Germany

*Correspondence: matthias.gimpel@tu-berlin.de; Tel.: +49 (0)30 314 79471





**Figure S1. Profiles of dissolved oxygen concentration, pH and biomass of the 2^nd^ preculture in EnPresso B medium.**

The 2^nd^ preculture was conducted in a 1-L single-use polycarbonate sensor flask with integrated sensors which allows on-line monitoring of pH, DO and optical biomass measurement. 150 mL EnPresso B medium and 3 U L^-1^ glucose-releasing reagent A were used in the 2^nd^ preculture shaken at 30 ℃ and 200 rpm for 20 h reaching an OD_600_ of about 7.





**Figure S2.** **Supplementary information of the fed-batch bioreactor cultivations.**

(A) Profiles of *on-line* dissolved oxygen (DO), pH, and applied stirrer speed (rpm) and volumetric oxygen transfer coefficient (*k_L_a*) determined from exhaust gas analysis data collected from the DO sensor. (B) Glucose concentration profiles for consumption including glucose initially added in the batch phase and fed glucose in the fed-batch phase and biomass yield coefficient (Y_X/S_) profiles estimated with how much biomass is produced per consumed glucose. (C) Profiles of oxygen uptake rate (Q_O2_) and carbon dioxide production rate (Q_CO2_) were estimated based on the inlet and outlet gas analysis of the bioreactor.


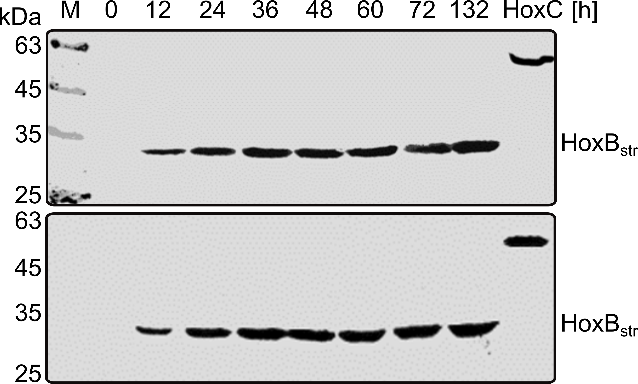


**Figure S3.** **Western Blot analysis of total RH production during the fed-batch bioreactor fermentation.**

1 mL cells were collected at different induction times from two parallelized bioreactor under the same conditions. Cells were normalized to OD_600_ 25 and 12 µL total protein samples were used on 12% polyacrylamide gels followed by Western blotting (WB). HoxB from RH was detected on the WB using anti-Strep tag antibody. 0.5 µg HoxC_str_ was used as control on the SDS-PAA gels.


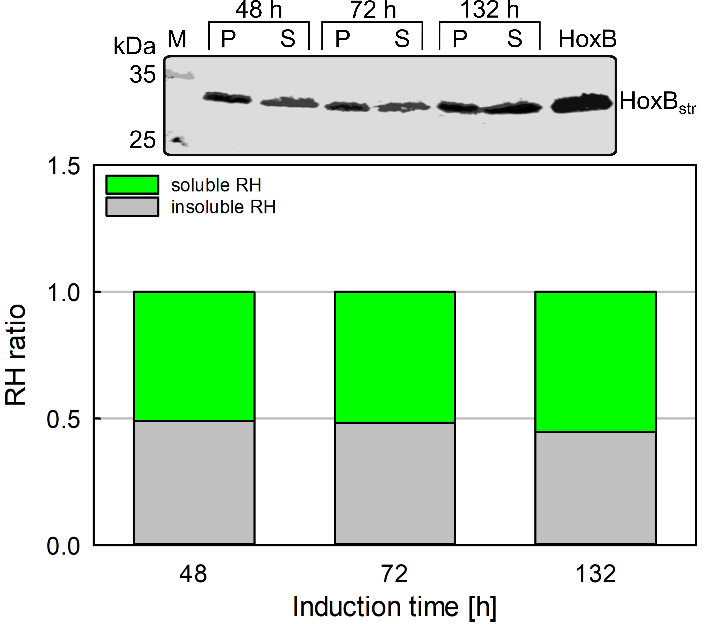


**Figure S4.** **Soluble and insoluble fractions of RH with Western blotting analysis.**

20 mL cells were collected at different induction times from two parallelized bioreactsor under the same conditions. Cells were disrupted with sonication on ice (60% amplitude, 7 mm sonotrode, 30 s on/off, 10 min) followed by centrifugation (8000 x*g*, 4 ℃, 90 min). The clarified lysates were purified using Strep-Tactin columns (CV of 500 µL). The soluble and insoluble protein fractions were analysed on Western Blots after sonication.
